# Context-Dependent Effects of HIV Disclosure on Social Isolation Among Rural PLHIV: A Pilot Configurational Study

**DOI:** 10.3390/ijerph22101480

**Published:** 2025-09-25

**Authors:** John Matta, Jacob Grubb

**Affiliations:** School of Engineering, Southern Illinois University Edwardsville, Edwardsville, IL 62026, USA

**Keywords:** disclosure behavior, HIV stigma, qualitative comparative analysis, rural health, social isolation

## Abstract

Social isolation is a critical but understudied concern for people living with HIV (PLHIV), particularly in rural U.S. communities where social visibility is high and access to supportive services is limited. Disclosure of HIV status is often framed as a health-promoting behavior that facilitates engagement with care and access to social support, yet it can also increase vulnerability to exclusion and isolation, especially where confidentiality is difficult to maintain. Using data from a pilot survey of rural PLHIV in the United States (n=17), this study examines when disclosure may function adaptively and when it may coincide with a heightened social burden. A Social Isolation Index was constructed from 15 indicators of exclusion across family, community, and institutional domains. Disclosure was measured both by the number of people informed and whether sexual partners were told. Typological methods and Qualitative Comparative Analysis (QCA) were applied to explore how disclosure patterns relate to race, sexual identity, and reported isolation. The results indicate that disclosure is not uniformly protective: several participants who disclosed widely also reported high levels of isolation, with heterosexual and Black participants often reporting a higher cumulative burden. These findings challenge one-size-fits-all assumptions about disclosure in public health messaging and underscore the need for tailored strategies that recognize both disclosure and nondisclosure as potentially adaptive responses in rural and marginalized communities.

## 1. Introduction

Social isolation is an important but underexamined public health concern for people living with HIV (PLHIV), particularly in rural U.S. communities where social visibility is high and access to supportive services is limited. Isolation can arise from multiple sources, including stigma, structural disadvantage, and limited access to social networks. According to GLAAD’s 2024 State of HIV Stigma Report, 85% of Americans still perceive stigma around HIV, with rural regions such as the Midwest and South identified as persistent areas of concern [1]. These contexts can magnify the challenges of maintaining privacy and finding social support.

Disclosure of HIV status is often framed as a health-promoting behavior that facilitates engagement in care and access to social resources [2,3], but it can also increase vulnerability to social isolation and exclusion [4,5], especially where confidentiality is harder to maintain. Qualitative studies in U.S. rural settings suggest that nondisclosure can sometimes function as a protective strategy when the risks of exposure, judgment, or rejection outweigh potential benefits, even in the absence of overt enacted stigma [6]. These findings underscore the complexity of disclosure decisions and highlight the need to account for both interpersonal and structural contexts.

Despite recognition of these dynamics, relatively little research has examined how disclosure patterns relate to experiences of social isolation in rural U.S. settings. Small-*N*, case-oriented methods remain underutilized for studying these complex configurational patterns. Qualitative Comparative Analysis (QCA) provides a way to explore how multiple factors interact to produce diverse outcomes [7]. This pilot study addresses this gap, using typological methods and QCA to investigate how disclosure behaviors, sexual identity, and race intersect with reported social isolation and related experiences in a small (n=17) survey of PLHIV in southern Illinois, USA.

We approach disclosure as a context-dependent, relational process and defer detailed theoretical discussion to the conceptual framework. Our goal is to explore the conditions under which disclosure may function adaptively or maladaptively, generating hypotheses that can inform more responsive interventions for rural and structurally marginalized populations.

Our research is guided by the following questions:

- How do HIV disclosure patterns among rural PLHIV relate to their experiences of social isolation and broader social burden?
- Under what conditions does disclosure appear adaptive (associated with lower isolation or exclusion), and when does it appear maladaptive (associated with higher isolation or exclusion)?
- How do these patterns differ by sexual identity (LGB vs. straight) and race (Black vs. White)?

With these goals in mind, we construct a Social Isolation Index and classify disclosure behaviors to examine how disclosure and isolation patterns intersect. This exploratory approach leverages small-*N*, qualitative–comparative methods to uncover configurational patterns rather than generalizable statistical trends.

## 2. Conceptual Framework

For PLHIV, disclosure occurs within broader social and structural contexts that shape access to support and vulnerability to exclusion. In rural areas, these dynamics are often intensified by limited anonymity, small social networks, and fewer available resources [8,9,10,11]. As a result, disclosure may lead to greater connection in some cases, but in others it may coincide with heightened social isolation.

We conceptualize disclosure as a relational process rather than a single act, embedded within overlapping social, cultural, and institutional conditions. Individual outcomes following disclosure depend on perceived safety, local stigma environments, and the availability of supportive networks. Prior qualitative studies in U.S. rural settings demonstrate that nondisclosure can be a strategic, protective choice, with individuals selectively concealing their status to avoid anticipated rejection, gossip, or social exclusion [6]. These findings underscore that disclosure cannot be understood solely in terms of personal preferences or attitudes, but must be situated within broader social constraints and vulnerabilities.

We draw on intersectionality as a sensitizing lens rather than a formal analytic framework [12,13]. Intersectionality highlights how systems of inequality, including racism, heterosexism, and institutional stigma, can combine to shape experiences of both disclosure and isolation. Given our small-*N* design, we do not test formal intersectional hypotheses, but we remain attentive to potential differences across overlapping dimensions of race and sexual identity.

Although we did not conduct formal intersectional analyses, the perspective of intersectionality nevertheless shaped both the design and interpretation of the study. In developing the typology and QCA truth tables, we included race and sexual identity alongside disclosure variables, because intersectional frameworks emphasize how overlapping systems of inequality (e.g., racism and heterosexism) may jointly influence outcomes for PLHIV. Similarly, in interpreting the results, we attended to subgroup differences, not to make definitive intersectional claims given the small sample, but to acknowledge that disclosure can have distinct social consequences depending on participants’ positions within multiple social hierarchies.

**Figure 1:**
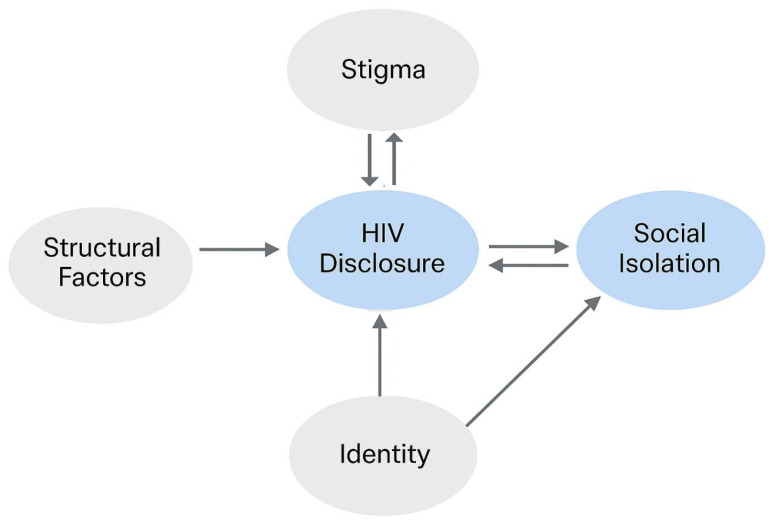
Conceptual framework illustrating how HIV disclosure is shaped by identity and structural factors, and how it, in turn, affects and is affected by social isolation. Arrows indicate hypothesized directions of influence.

Figure 1 illustrates our framework. At its center, HIV disclosure is conceptualized as a dynamic process influenced by personal factors, anticipated outcomes, and broader structural factors. Arrows indicate hypothesized influences and feedback loops, recognizing that disclosure choices can affect, and be affected by, experiences of social burden and support.

Finally, our framework extends the Disclosure Processes Model (DPM) [14], which views disclosure as a context-sensitive decision informed by anticipated benefits, personal attributes, and environmental constraints. We build on the DPM by explicitly integrating structural factors, including rurality, institutional stigma, and overlapping social identities, while recognizing that our pilot sample size limits deeper statistical exploration of these intersections.

In summary, this framework guides our exploratory analysis by emphasizing that disclosure is neither inherently adaptive nor maladaptive. Its consequences emerge from the interaction between individual choices and broader contexts of social isolation, support, and exclusion.

## 3. Related Work

Research on HIV disclosure has long emphasized its dual nature: disclosure can lead to beneficial outcomes such as increased social support and better care engagement, but it can also expose individuals to rejection, discrimination, and emotional harm [14,15,16]. However, much of this work has focused on urban or clinical contexts or relied on large-*N* survey methods [17], often overlooking how disclosure operates within small, rural samples or among multiply marginalized individuals. Several studies have documented that disclosure can be maladaptive in environments characterized by high levels of stigma or limited social support [18,19].

Research has identified multiple barriers in rural settings, including limited confidentiality, geographic isolation, and a shortage of culturally competent healthcare providers [11,20]. Turan et al. conceptualize stigma as a multilevel phenomenon encompassing interpersonal, community, and structural domains, which together produce a cumulative burden for individuals in marginalized contexts [18]. These structural and contextual factors are associated with elevated risks of social isolation, particularly among individuals occupying multiple marginalized social positions.

While not being directly used in this work, intersectionality provides a critical framework for understanding how overlapping social identities, such as race, sexuality, and rurality, shape health experiences and outcomes [12]. Research has documented that Black and LGBT individuals living with HIV often encounter stigma both within their identity communities and in broader social contexts [21,22]. These intersecting forms of vulnerability may influence how disclosure is perceived and received, thereby shaping whether it results in social support or further marginalization. This work builds on prior work by the current author, which examined the intersectionality of HIV-related discrimination [23].

QCA has been utilized in both small and complex populations to identify combinations of conditions with particular outcomes, providing a structured methodology to examine phenomena such as stigma and disclosure [24,25]. Similarly, typological approaches support the classification of heterogeneous response patterns, particularly in exploratory or pilot datasets [26], such as the pilot dataset our research is based upon.

## 4. Methods

This study employs a mixed descriptive and configurational approach to examine the relationship between HIV disclosure and social isolation among PLHIV in a rural setting. Using data from a pilot survey, we combined small-*N* case-based analysis with typological methods and QCA to identify intersecting patterns of vulnerability and resilience. The methodological emphasis was on understanding how disclosure behaviors interact with social identity, particularly race and sexual orientation, within a high-stigma, under-resourced context.

### 4.1. Study Design and Data Collection

This research draws on the *Burden of HIV* survey, a cross-sectional pilot study conducted between 2021 and 2023 in southern Illinois, as previously described in [23,27] and referenced in [28]. The survey was designed to explore the intersection of social determinants of health (SDoH), stigma, and structural vulnerability among PLHIV in a rural U.S. region.

Survey participants (n=22) were recruited through HIV service providers and community outreach in trusted locations, such as doctor’s offices and medical clinics, using a modified respondent-driven sampling (RDS) approach. Respondents were given referral slips to recruit other potential respondents, allowing for better coverage and access to individuals otherwise considered hard-to-reach by traditional sampling methods. Surveys were completed online. Eligibility requirements included an age of 18 or older and membership of one or more of the following categories: people living with HIV (PLHIV), men who have sex with men (MSM), people who inject drugs (PWID), or sexual/romantic partners of these groups. Purposive recruitment ensured diversity by race, gender identity, sexual orientation, and socioeconomic background.

Among the survey participants, 17 were PLHIV (upon which this study is based), and 8 identified as MSM. The participants included 13 cisgender men, 5 cisgender women, and 4 non-binary or transgender individuals. By race and ethnicity, 15 identified as Black, 4 as White, and 3 as Hispanic. Regarding sexual orientation, 11 participants identified as gay or lesbian, 4 as bisexual, and 5 as straight.

The survey was administered by the Applied Research Consultants (ARC) at Southern Illinois University Carbondale. All recruitment and survey materials and procedures were approved with strict oversight from the SIUE IRB. The use of RDS with participant-driven recruitment, when combined with ethical oversight from institutional review boards and informed consent from participants, has been shown to respect ethical boundaries, while increasing participation by hard-to-reach groups [29]. Participants received USD 10 in compensation for their response and an optional additional USD 10 each for up to three referrals. All data were de-identified prior to analysis.

### 4.2. Key Measures and Index Construction

#### 4.2.1. Disclosure Variables

Disclosure was operationalized using two binary measures which are referenced throughout this study:

- InformedSexPartners: Whether the respondent had consistently disclosed their HIV status to sexual partners.
- Told3Plus: Whether the respondent had disclosed to three or more individuals. While no universal threshold exists, prior qualitative studies suggest that disclosure to three or more confidants signals a shift toward openness and support-seeking behavior [14,18]. This threshold was used as an exploratory but conceptually grounded measure of high disclosure.

#### 4.2.2. Social Isolation Index

To capture cumulative exclusion, we constructed a Social Isolation Index based on 15 binary survey question categories covering interpersonal rejection (e.g., “I feel like I have no one to turn to,” “Friends are distant after revealing my HIV status”), identity-based exclusion (e.g., “I feel part of the LGBT community”), and institutional mistreatment (e.g., “I am treated unfairly at work”). While not indicating direct causation, a correlation between these categories and disclosure variables can be seen. These social isolation items were one-hot encoded and summed into a continuous Social Isolation Score (range: 0–15). A binary variable, SocialIsolationIndex, was then created to indicate whether the participant scored 7 or higher, reflecting above-average isolation based on the sample mean. Using this threshold, 10 out of 17 participants (59%) were categorized as socially isolated.

### 4.3. Analytical Approach

This research uses a three-part strategy to explore patterns between disclosure variables and the Social Isolation Index: Visual Pattern Exploration, Typology Construction, and QCA Configurational Analysis.

#### 4.3.1. Visual Pattern Exploration

Initial visual analyses were used to explore potential relationships between HIV disclosure and social isolation. A scatterplot was constructed to compare the number of people to whom participants disclosed their HIV status against their Social Isolation Index score. This was followed by a typological matrix classifying participants based on high or low levels of disclosure and isolation. Additional visualizations, including a radar plot of multidimensional burden and a heatmap of stigma indicators by identity and disclosure category, were incorporated later in the analysis to highlight domain-specific forms of exclusion. These visual tools supported the identification of emergent patterns and informed the development of typologies and QCA configurations.

#### 4.3.2. Typology Construction

Participants were classified into one of four typology groups based on their disclosure and isolation status. This typology matrix enabled visual and categorical pattern identification across social identity groups and social isolation experiences. We tested the robustness of these groupings using an alternate disclosure threshold (see Section 5.8).

#### 4.3.3. QCA Configuration Analysis

Crisp-set QCA, in which all conditions and outcomes are coded as binary variables (i.e., present = 1, absent = 0), was used to explore how disclosure behavior, sexual orientation, and race interacted to predict the presence or absence of specific social isolation outcomes. We chose crisp-set QCA due to the binary nature of our data and small sample size, as it allows clear identification of specific configurations without the ambiguity introduced by the partial membership scores that are common in fuzzy-set QCA. Most key conditions, such as disclosure behavior, sexual identity, and social isolation experiences, were recorded as dichotomous variables, making crisp-set analysis the most appropriate configurational approach for this dataset.

Binary truth tables were constructed from conjunctural conditions and used to identify consistent configurations associated with five key experiences: family rejection, friend rejection, name-calling, unfair treatment at work, and relocation due to stigma.

To ensure methodological rigor, we followed best practices for small-*N* QCA applications. These included the following:

- Limiting the number of causal conditions per model to avoid overfitting.
- Basing variable selection on both theoretical relevance and patterns observed in preliminary visual analysis.
- Using a minimum frequency threshold of one for configurations to be considered.
- Interpreting results cautiously in light of the sample size and the exploratory nature of the study.

QCA was selected for its ability to detect situations in which multiple conditions combine to produce an outcome and for its suitability to studies with small but conceptually rich datasets.

Taken together, this multi-method approach allowed for an integrated analysis of how disclosure behavior and social identity relate to social isolation in a rural context. Typological and configurational techniques were used to identify patterns of adaptive and maladaptive disclosure, while visual tools supported interpretation of complex relationships across multiple domains. The results that follow are presented in a stepwise fashion, beginning with descriptive trends, followed by typological patterns, and culminating in QCA truth tables and domain-specific burden profiles.

## 5. Results

This pilot study explores patterns of HIV disclosure, social isolation, and related social burdens among a small sample of rural PLHIV. Given the limited sample size (n=17), the analyses are exploratory and descriptive rather than confirmatory. The goal is not to test causal hypotheses, but to identify potential patterns that may inform future research.

Throughout, we emphasize that these findings should be interpreted cautiously. The small-*N* design and uneven subgroup sizes limit generalizability, and observed associations may reflect multiple unmeasured factors. We frame these results as a first step toward understanding how disclosure, social isolation, and identity interact within rural contexts.

### 5.1. Disclosure and Social Isolation: Exploratory Scatterplot

**Figure 2:**
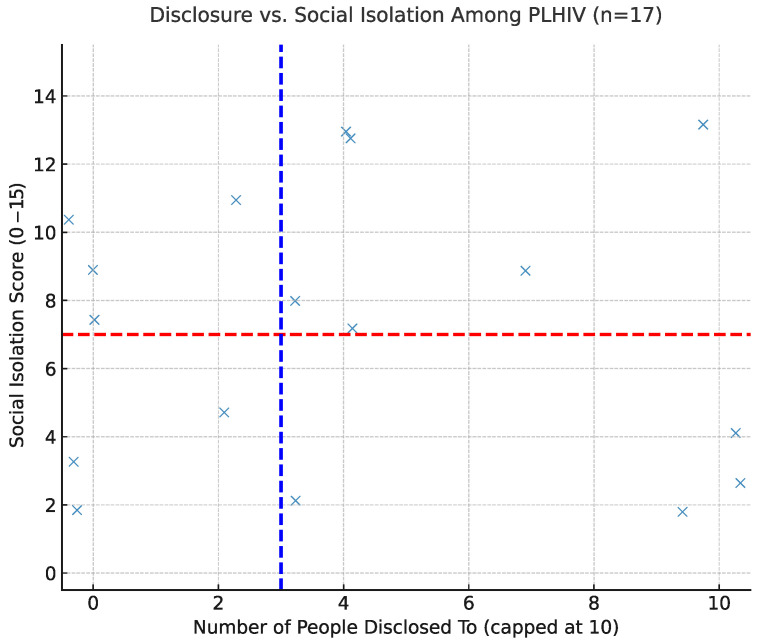
Disclosure vs. social isolation among HIV-positive participants. Each point represents one participant. Horizontal red line = isolation threshold (7); vertical blue line = sample median number of disclosures. Jitter added for readability.

We created a scatterplot in which each point represents a PLHIV survey respondent (Figure 2, n=17). The x-axis shows the number of people to whom the participant disclosed their HIV status, drawn from the PeopleToldHIV variable and capped at 10 for interpretability. The y-axis reflects the cumulative number of distinct social isolation experiences, calculated as the sum of 15 binary indicators (e.g., family rejection, lack of support, verbal abuse).

To minimize point overlap, random jitter was applied. Dashed reference lines denote the sample median for disclosure (blue) and the isolation threshold of 7 (red), consistent with the construction of the Social Isolation Index.

The scatterplot reveals substantial heterogeneity: participants with high disclosure appear across the full range of social isolation, and those with low disclosure also report both low and high isolation. In other words, greater openness about HIV status does not consistently predict either a lower or higher social burden in this sample.

These findings align with prior qualitative studies showing that the consequences of disclosure can vary considerably depending on individual circumstances and social context, particularly in rural settings. Because of the small sample size, these results should be viewed as descriptive rather than definitive.

### 5.2. Typology of Disclosure and Isolation

**Figure 3:**
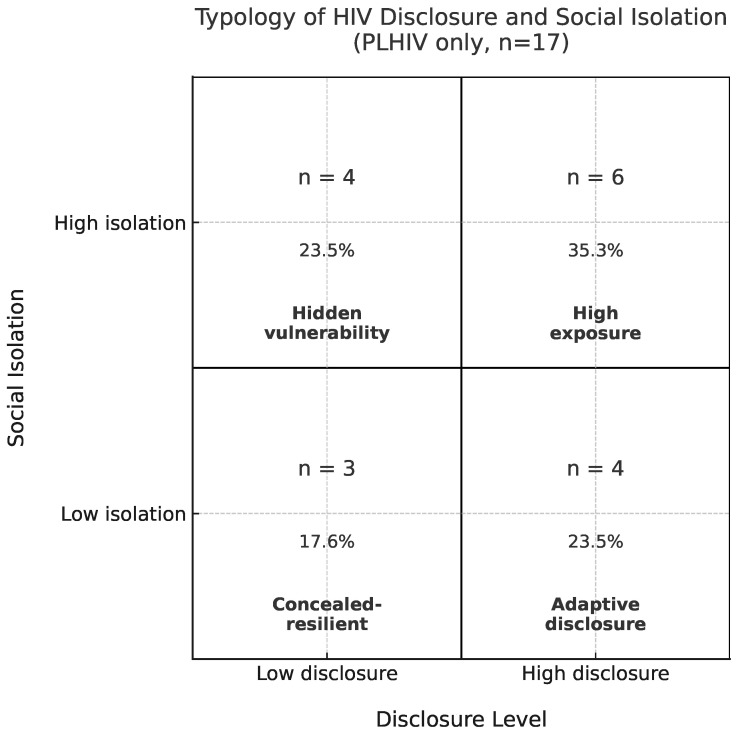
Typology of HIV disclosure and social isolation. Disclosure defined as Told3Plus (≥3 people). Isolation defined as 7 or more reported exclusion experiences. Each quadrant shows label, count, and percentage of participants (n=17).

To explore patterns linking disclosure and social isolation, we classified PLHIV participants into four interpretive categories based on their relative positions in the disclosure–isolation space (Figure 3). These categories are used heuristically to describe broad tendencies in the data and should not be interpreted as definitive groupings, especially given the small sample size.

- Higher Disclosure/Lower Isolation (“Adaptive Disclosure”): Among this small subgroup of four, participants reported disclosing to many individuals while also experiencing relatively low levels of social burden. In this sample, openness co-occurs with lower reported isolation for these participants, though others with similar disclosure levels reported higher isolation.
- Higher Disclosure/Higher Isolation (“High Exposure”): Some participants (n=6) disclosed widely and also reported an elevated social burden. This co-occurrence could reflect several possibilities, including stigma, pre-existing social exclusion, or other unmeasured factors. These data cannot distinguish among these explanations.
- Lower Disclosure/Lower Isolation (“Concealed/Resilient”): Three participants disclosed selectively or minimally, yet did not report high levels of social burden. Selective nondisclosure may coincide with lower reported isolation for these individuals, but this pattern is not consistent across the sample.
- Lower Disclosure/Higher Isolation (“Hidden Vulnerability”): Four participants disclosed to few or no individuals, yet reported relatively high isolation. This could indicate that nondisclosure alone is insufficient to prevent social exclusion, or it may reflect pre-existing isolation. Given the very small number of participants in this category, these patterns should be interpreted cautiously.

Overall, these patterns suggest no single, linear relationship between disclosure and social isolation. Individuals with similar disclosure counts reported markedly different levels of social burden, underscoring the variability in participants’ experiences. These findings are exploratory and descriptive, highlighting possible tendencies in this pilot sample, rather than establishing causal relationships.

### 5.3. Cumulative Social Burden by Disclosure Pattern

To extend the typology analysis, we calculated a cumulative burden score by summing the 15 binary indicators of social exclusion for each participant. This composite score represents the total number of distinct burden experiences across interpersonal, community, and institutional domains. Analyses were restricted to participants living with HIV.

**Figure 4:**
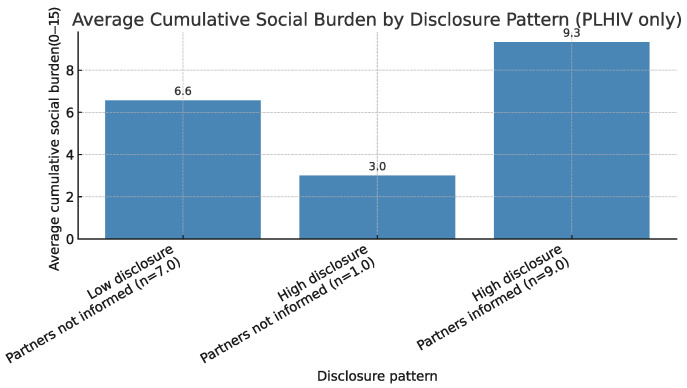
Average cumulative social burden (sum of 15 exclusion indicators) by disclosure pattern among PLHIV (n=17). Disclosure defined by Told3Plus and InformedSexPartners.

Figure 4 displays the mean cumulative burden for groups defined by two disclosure dimensions: whether participants disclosed to three or more individuals (Told3Plus) and whether they informed their sexual partners (InformedSexPartners). These groupings distinguish both the breadth and relational specificity of disclosure.

Among the 17 PLHIV, participants who disclosed broadly and informed their sexual partners (n=9) reported the highest average cumulative burden (mean =9.3 out of 15 possible indicators). Those who disclosed to fewer than three individuals and did not inform partners (n=7) reported a lower mean burden (mean =6.6). Only one participant disclosed widely but did not inform partners (n=1, mean =3.0). No participants reported low overall disclosure while informing their partners.

These results suggest that broader disclosure may coincide with greater exposure to social exclusion in some contexts, although nondisclosure is not uniformly protective. Because subgroup sizes are small, these findings are descriptive and should be interpreted with caution.

### 5.4. Multidimensional Social Burden by Disclosure Group

**Figure 5:**
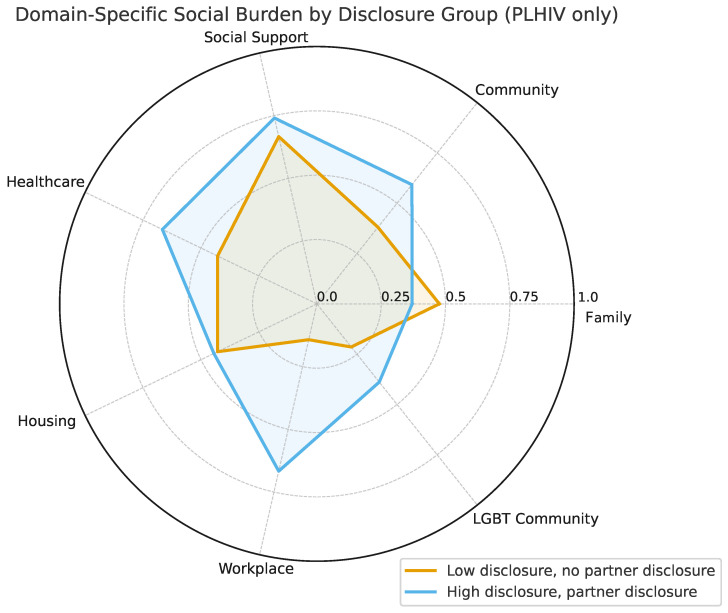
Radar plot showing mean reported social burden across seven domains by disclosure group. Two groups are shown: those who disclosed to fewer than three people and did not inform sexual partners (n=7), and those who disclosed to three or more people and informed partners (n=9). Higher values indicate greater reported burden in that domain. Subgroup sizes are small; results are descriptive.

To examine how HIV disclosure relates to different forms of reported social burden, we disaggregated the 15 binary indicators into seven thematic domains: Family, Community, Social Support, Healthcare, Housing, Workplace, and LGBT Community. For each domain, participants’ responses were averaged to create a domain-specific burden score. These scores were visualized using a radar chart (Figure 5) to compare multidimensional isolation profiles across disclosure groups.

Two primary disclosure groups are shown:

- Low Disclosure, No Partner Disclosure (n=7, 41%): Participants who disclosed to fewer than three people and did not inform their sexual partners.
- High Disclosure, Partner Disclosure (n=9, 53%): Participants who disclosed to three or more individuals and informed their sexual partners.

Only these two groups were visualized, because they represented the dominant configurations among HIV-positive participants. One additional disclosure pattern (disclosed widely but did not inform partners) was rare (n=1), and was omitted for clarity. No participants disclosed to fewer than three people while informing their partners.

The radar plot highlights considerable variability across social domains. Participants in the Low Disclosure, No Partner Disclosure group reported a higher or comparable average burden in many domains, particularly in family-related exclusion, although they reported a lower burden in the workplace and community. By contrast, participants in the High Disclosure, Partner Disclosure group reported a somewhat greater burden in healthcare-, community-, and workplace-related items. However, given the small sample size and uneven subgroup counts, these differences should be interpreted cautiously.

### 5.5. Profiles of Reported Social Burden by Disclosure and Identity

**Figure 6:**
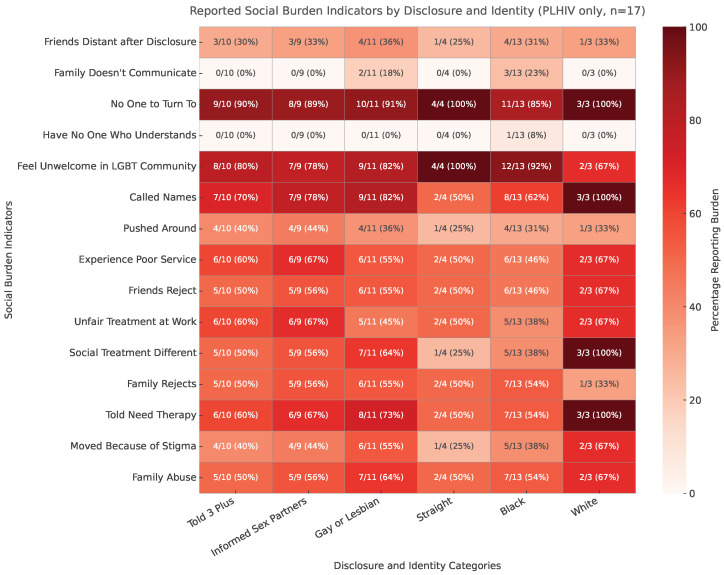
Reported social burden indicators by disclosure and identity among rural PLHIV (n=17). The heatmap shows the percentage of participants reporting each of 15 exclusion-related experiences, with raw counts and percentages annotated in each cell. Darker shading indicates higher reported burden.

To examine how specific types of social burden vary across disclosure and identity groups, we constructed a heatmap (PLHIV only, n=17) summarizing the proportion of participants reporting each of 15 exclusion-related experiences across six categories: Told3Plus, InformedSexPartners, GayOrLesbian, Straight, Black, and White (Figure 6). Each cell displays the raw count and percentage for reference.

Among participants who disclosed to three or more people (Told3Plus = 1; n=10), 7/10 (70%) reported being called names, 5/10 (50%) reported family rejection, and 6/10 (60%) reported unfair treatment at work. Those who informed sexual partners (InformedSexPartners = 1; n=9) showed similar or higher levels: 7/9 (78%) were called names, 5/9 (56%) suffered family rejection, and 6/9 (67%) experienced workplace unfairness.

Identity-based patterns were also evident. Among gay and lesbian participants (n=11), a large majority reported verbal harassment (9/11, 82%) and more than half experienced family rejection (6/11, 55%). In contrast, straight participants (n=4) showed more moderate but consistent levels of burden, with about half reporting each of these experiences. By race, Black participants (n=13) frequently reported being called names (8/13, 62%) and family rejection (7/13, 54%), while White participants (n=3) reported high levels of verbal harassment (3/3, 100%) but variable burden in other domains. Because the subgroup sizes for straight and White participants are small, these estimates should be interpreted cautiously.

Overall, the heatmap highlights the multidimensional nature of social burden among rural PLHIV. Disclosure, race, and sexual identity each shape vulnerability profiles, but no single identity or disclosure pattern is uniformly protective. These descriptive patterns underscore the importance of context when evaluating the social consequences of disclosure.

### 5.6. Social Burden Outcomes by Disclosure Pattern and Sexual Identity

To examine how disclosure behavior and sexual identity combine to shape reported experiences of exclusion, we used Qualitative Comparative Analysis (QCA) to summarize five indicators: family rejection, friend rejection, verbal harassment (being called names), unfair treatment at work or school, and moving due to stigma. QCA identifies patterns of conditions that consistently align with particular outcomes, making it well-suited for exploring configurational relationships in small samples.

The results are presented in a truth table format (Table 1). Each row represents one observed configuration across four binary conditions: Told3Plus, InformedSexPartners, LGB, and Straight. For each configuration, we report the subgroup size and the percentage (with raw count) of participants endorsing each outcome. The subgroup sizes are very small, so these findings should be interpreted descriptively.

Several tentative tendencies emerged:

- High disclosure among straight participants (n=2): In this subgroup, 100% reported friend rejection, verbal harassment, and workplace discrimination; half reported moving because of stigma. While striking, these findings reflect only two participants and should be interpreted cautiously.
- High disclosure among LGB participants (n=7): Participants who disclosed widely and identified as LGB reported a slightly lower average burden than their straight counterparts. For example, 71.4% reported being called names, 57.1% reported workplace mistreatment, and 42.9% reported moving due to stigma.
- Low disclosure among LGB participants (n=6): Even when disclosure was limited, moderate levels of reported burden persisted. In this group, 66.7% reported being called names, 50% reported family rejection, and 50% reported having moved because of stigma.

Overall, these patterns illustrate that no single disclosure strategy or identity group is uniformly associated with a lower reported burden. Observed co-occurrences of disclosure, identity, and exclusion experiences likely reflect unmeasured social and structural factors. Because the subgroup sizes are very small and these analyses are exploratory, these results should be considered preliminary and hypothesis-generating.

### 5.7. Racial Differences in Social Burden Following Disclosure

To examine how race moderates the relationship between disclosure behavior and reported experiences of exclusion, we recalculated the analysis using race instead of sexual orientation (Table 2). Each row reflects one observed configuration across four binary conditions: Told3Plus, InformedSexPartners, Black, and White. For each configuration, we report the subgroup size and the percentage (with raw count) of participants endorsing each indicator.

Across disclosure profiles, racial disparities emerged. Among participants who disclosed widely and informed their partners, all White participants (n=2) reported being called names and experiencing workplace discrimination, with 50% also reporting family rejection and moving due to stigma. Black participants in this same disclosure configuration (n=6) also reported high rates: 83.3% were called names, 66.7% experienced workplace discrimination, and 33.3% had moved due to stigma. These findings suggest that full disclosure exposes both groups to substantial social risks.

Stigma and isolation burdens were also evident among low-disclosure participants. Among those who disclosed to few people and did not inform partners, 50% of Black participants (n=6) reported family rejection, 50% reported moving, and 50% reported being called names. The sole White participant in this category reported both friend rejection and verbal harassment. While conclusions are limited by the small subgroup sizes, these patterns highlight that nondisclosure does not uniformly protect against a reported burden, particularly for Black PLHIV.

### 5.8. Robustness Checks (Summary)

To assess the stability of our findings, we conducted a series of robustness checks by varying the disclosure and isolation thresholds used in the typology and QCA analyses. For the typology, using stricter thresholds (Told4Plus and a Social Isolation Index cutoff of ≥8) shifted participants into only two categories, Adaptive Disclosure and Concealed/Resilient, with no participants meeting criteria for High Disclosure or Hidden Vulnerability. This indicates that, in a small sample, the distribution of typology categories is sensitive to threshold definitions, though participants’ relative positions remain stable.

For the QCA analyses, subgroup percentages shifted somewhat under stricter disclosure thresholds, particularly for configurations with few participants. However, broader configurational patterns were consistent: participants with high disclosure and a straight identity tended to report the highest burden, while nondisclosure was not uniformly protective, especially among Black participants and those with overlapping marginalized identities.

Detailed robustness tables and full interpretations are provided in Appendix A.

## 6. Discussion

This pilot study examined how HIV disclosure patterns relate to reported social burden among rural PLHIV. Across analyses, disclosure was neither uniformly adaptive nor maladaptive. Instead, outcomes varied depending on context, identity, and the domains of social life where exclusion occurred. For some participants, greater openness coincided with a lower reported burden, while others with similar disclosure patterns experienced higher isolation and exclusion (Figure 2). These findings underscore the need to move beyond binary assumptions of “disclosure as good” versus “nondisclosure as bad” and instead focus on the conditions under which disclosure may be protective or harmful.

Our findings build on and extend theoretical models of HIV disclosure and social burden. The Disclosure Processes Model (DPM) emphasizes how disclosure outcomes depend on anticipated reactions, available support, and environmental context [14], while stigma frameworks distinguish between anticipated, enacted, and internalized stigma [30]. Consistent with these theories, our results demonstrate that disclosure is not uniformly adaptive: for some participants, greater openness coincided with elevated isolation and burden, patterns that may be amplified in rural contexts where close-knit social networks limit anonymity.

Recent U.S.-based research supports these findings. Quinn et al. (2020) show that rural older PLHIV often face compounding stigma and social isolation, contributing to heightened depression and loneliness [31]. Walsh et al. (2023) further demonstrate that among rural older PLHIV, low social support and high perceived stress are strongly linked to poorer quality of life [32]. Similarly, Gervolino, Krause, and Halkitis report that fragmented social networks among older adults living with HIV undermine well-being and increase vulnerability to mental health challenges [33]. Together, these studies highlight that disclosure occurs within overlapping social and structural vulnerabilities where its effects are deeply context-dependent.

We also engaged intersectionality as a guiding framework for design and interpretation. Although our small sample precluded formal intersectional analyses, the typology and QCA explicitly incorporated race and sexual identity, reflecting how overlapping social positions can shape disclosure’s consequences. The descriptive subgroup differences observed in our results suggest that compounded marginalizations, such as being both Black and living with HIV in a rural community, may intensify the social burdens of disclosure. Future research should use larger, more diverse samples and explicitly intersectional designs to clarify how multiple social identities interact with structural conditions to produce varied disclosure outcomes.

Public health messaging often frames disclosure as inherently beneficial. However, our findings add to evidence showing that disclosure can coincide with rejection, harassment, and institutional mistreatment [4,13,34]. For example, among participants who disclosed to three or more people (Told3Plus, n=10), 7/10 (70%) reported being called names, 5/10 (50%) reported family rejection, and 6/10 (60%) reported unfair treatment at work (Figure 6). These results mirror patterns seen in prior studies [6] and suggest that disclosure guidance must be context-specific, particularly in rural settings.

One of the most striking patterns appears in the “high-disclosure/high-burden” configurations (Figure 3 and Figure 4). Several explanations are plausible:

- Pre-existing isolation: Some participants may have been socially isolated before disclosure; openness did not alleviate existing exclusion.
- Local stigma environments: In small rural communities, disclosure can increase exposure to gossip, social rejection, or workplace mistreatment, consistent with Preston et al. [35].
- Identity-related vulnerabilities: Participants holding marginalized identities (e.g., Black PLHIV or LGB individuals) may experience layered social burdens unrelated to disclosure itself, consistent with Jeffries et al. [13].

Because the subgroup sizes are small, these findings are descriptive rather than causal. However, the recurrence of high-disclosure/high-burden patterns across studies suggests that disclosure strategies should be tailored to individual and contextual factors, rather than promoted uniformly.

These findings have several implications. First, public health messaging should avoid assuming that disclosure is always beneficial. Interventions must recognize that disclosure decisions are embedded within structural and interpersonal contexts that can magnify or mitigate risk. Second, future research should use larger, more diverse samples to test these exploratory findings. Finally, our results underscore the value of treating disclosure as a relational, context-dependent process rather than a universal public health good.

The findings also suggest that disclosure-support strategies should be tailored to individuals’ identities, contexts, and available resources, particularly in rural settings. For example, peer navigator programs have improved HIV care engagement and reduced isolation among PLHIV in rural Alabama [36]. Similarly, mobile health tools like the Positive Links platform provide individualized disclosure counseling and virtual support for rural PLHIV [37]. Structural interventions such as CDC’s Let’s Stop HIV Together campaign aim to reduce stigma at the community level, while culturally responsive LGBTQ+ support networks, such as those provided by the Southern AIDS Coalition, address overlapping burdens for marginalized identity groups. Together, these examples illustrate how public health strategies can operationalize identity-sensitive, context-specific approaches to disclosure.

## 7. Conclusions

This pilot study examined how HIV disclosure intersects with race, sexual identity, and rural context to shape reported social isolation and burden among PLHIV. Across multiple analyses, disclosure was neither uniformly adaptive nor maladaptive: for some, openness coincided with lower isolation, while for others, similar disclosure patterns were associated with heightened exclusion. Importantly, nondisclosure was not consistently protective, particularly among participants facing overlapping vulnerabilities related to race or sexual identity.

These findings highlight the need to approach disclosure as a context-dependent relational process rather than a universal public health good. In rural settings where social networks are small and privacy is difficult to maintain, interventions and messaging should avoid one-size-fits-all recommendations and instead prioritize tailored strategies that account for identity, context, and available support systems. Future studies using larger, more diverse samples and mixed-methods designs are needed to clarify when disclosure is protective versus harmful and to inform interventions that reduce social burden while supporting care engagement.

## Figures and Tables

**Table 1:** Reported exclusion experiences by disclosure behavior and sexual identity (PLHIV only, n=17). Results are descriptive; subgroup sizes are small.

Told 3 Plus	Informed Partners	LGB	Straight	Count	Family Rejects	Friends Reject	Called Names	Work Unfair	Moved Because of Stigma
0	0	0	1	1	0 (0.0%)	0 (0.0%)	0 (0.0%)	0 (0.0%)	0 (0.0%)
0	0	1	0	6	3 (50.0%)	3 (50.0%)	4 (66.7%)	1 (16.7%)	3 (50.0%)
1	0	0	1	1	0 (0.0%)	0 (0.0%)	0 (0.0%)	0 (0.0%)	0 (0.0%)
1	1	0	1	2	2 (100.0%)	2 (100.0%)	2 (100.0%)	2 (100.0%)	1 (50.0%)
1	1	1	0	7	3 (42.9%)	3 (42.9%)	5 (71.4%)	4 (57.1%)	3 (42.9%)

**Table 2:** Reported exclusion experiences by disclosure behavior and race (PLHIV only, n=17). Results are descriptive; subgroup sizes are small.

Told 3 Plus	Informed Partners	Black	White	Count	Family Rejects	Friends Reject	Called Names	Work Unfair	Moved Because of Stigma
0	0	0	1	1	0 (0.0%)	1 (100.0%)	1 (100.0%)	0 (0.0%)	0 (0.0%)
0	0	1	0	6	3 (50.0%)	2 (33.3%)	3 (50.0%)	1 (16.7%)	3 (50.0%)
1	0	1	0	1	0 (0.0%)	0 (0.0%)	0 (0.0%)	0 (0.0%)	0 (0.0%)
1	1	0	0	1	0 (0.0%)	0 (0.0%)	0 (0.0%)	0 (0.0%)	0 (0.0%)
1	1	0	1	2	1 (50.0%)	1 (50.0%)	2 (100.0%)	2 (100.0%)	2 (100.0%)
1	1	1	0	6	4 (66.7%)	4 (66.7%)	5 (83.3%)	4 (66.7%)	2 (33.3%)

## Data Availability

The Burden of HIV survey results and analysis materials are freely available, and others are encouraged to use these data. The code used in the original study can be accessed at https://github.com/SIUEComplexNetworksLab/BOHComplexNetworks (accessed on 23 September 2025). Data are available at https://www.openicpsr.org/openicpsr/project/192186/version/V1/view (accessed on 23 September 2025).

## Abbreviations

The following abbreviations are used in this manuscript:

PLHIV	People Living with HIV
QCA	Qualitative Comparative Analysis
SI Index	Social Isolation Index
LGB	Lesbian, Gay, or Bisexual
Told3Plus	Disclosed HIV Status to Three or More People
Told4Plus	Disclosed HIV Status to Four or More People

## Appendix A. Robustness Checks: Threshold Sensitivity in Typology and QCA

To evaluate the stability of our findings, we conducted robustness checks by varying the disclosure and social isolation thresholds used in the typology and QCA analyses. Because the sample size is small (n=17 PLHIV), these checks help to clarify which observed patterns are stable across reasonable threshold definitions and which are more sensitive to analytic choices.

### Appendix A.1. Typology Robustness: Alternate Thresholds

To evaluate the stability of the typology classification, we re-ran the four-category typology using more conservative thresholds for both disclosure and social isolation. In the main analysis, *high disclosure* was defined as informing three or more people (Told3Plus), and *high isolation* was defined as reporting seven or more exclusion experiences on the Social Isolation Index.

For the robustness check, we used stricter thresholds: informing four or more people (Told4Plus) and reporting eight or more exclusion experiences.

The stricter cutoffs substantially shifted participants into only two categories:

- Adaptive Disclosure (n=8, 47.1%): Higher disclosure with lower isolation.
- Concealed/Resilient (n=9, 52.9%): Lower disclosure with lower isolation.

No participants met the criteria for High Disclosure or Hidden Vulnerability under these stricter thresholds. This result highlights how, with a small sample, typology structure is sensitive to threshold selection.

### Appendix A.2. QCA Sensitivity: Sexual Identity Configurations

We evaluated the sensitivity of QCA results to alternative disclosure thresholds using sexual identity configurations. In the main analysis, *high disclosure* was defined as informing three or more people (Told3Plus), whereas in the robustness check we used a stricter threshold of four or more (Told4Plus). Table A1 presents selected rows from the truth table, summarizing reported exclusion outcomes across four binary conditions: Told3Plus/Told4Plus, InformedSexPartners, LGB, and Straight. For each configuration, we show the percentage of participants endorsing four exclusion indicators: family rejection, being called names, workplace unfairness, and moving due to stigma.

Patterns were broadly consistent under both thresholds. Among participants who disclosed widely and informed partners, straight individuals (n=2) consistently reported the highest burden across all indicators: 100% experienced family rejection, verbal harassment, and workplace unfairness, and 50% moved due to stigma. LGB participants with similar disclosure patterns (n=7) reported slightly lower, but still elevated, levels of burden: 43% reported family rejection, 71% reported verbal harassment, and 57% reported workplace unfairness under the main threshold; under the stricter threshold, these levels rose modestly to 80% for being called names and 60% for workplace unfairness.

**Table A1:** QCA outcomes by disclosure threshold using sexual identity configurations (PLHIV only, n=17). Percentages shown for each burden indicator under Told3Plus/Told4Plus.

Told3+/4+	Informed Partners	LGB	Straight	Family Rejects	Called Names	Unfair Work	Moved (Stigma)
0	0	1	0	50%/50%	67%/67%	17%/17%	50%/50%
1	1	1	0	43%/43%	71%/80%	57%/60%	43%/40%
1	1	0	1	100%/100%	100%/100%	100%/100%	50%/50%

Overall, the QCA results are qualitatively stable across thresholds, but subgroup sizes are small, and individual outcomes vary. These findings reinforce the exploratory nature of the analysis and highlight the need for larger studies to confirm configurational patterns.

### Appendix A.3. QCA Sensitivity: Racial Identity Configurations

We also assessed the sensitivity of QCA results to disclosure thresholds using racial identity configurations. As with the previous analysis, *high disclosure* was defined in the main analysis as informing three or more people (Told3Plus), while the robustness check used a stricter threshold of four or more (Told4Plus). Table A2 shows selected configurations, summarizing reported exclusion outcomes across four binary conditions: Told3Plus/Told4Plus, InformedSexPartners, Black, and White. Percentages are reported for four exclusion indicators: family rejection, being called names, workplace unfairness, and moving due to stigma.

Under both thresholds, participants identifying as White (n=3) who disclosed widely and informed partners reported the highest levels of burden: 100% reported being called names, 50% reported family rejection, 100% reported workplace unfairness, and 100% reported having moved due to stigma. Black participants with similar disclosure patterns (n=6) also reported elevated burdens but at slightly lower levels: 67% reported being called names, 67% reported family rejection, and 33% reported having moved due to stigma under the main threshold.

Among the low-disclosure groups, substantial racial disparities emerged. Among black participants who disclosed to few people and did not inform partners (n=6), 50% reported family rejection, 50% reported verbal harassment, and 50% reported moving due to stigma. In contrast, White participants in the same low-disclosure category (n=1) reported no family rejection, but 100% reported verbal harassment. These findings suggest that nondisclosure is not uniformly protective, particularly for Black participants, who may face stigma-related experiences regardless of disclosure patterns.

**Table A2:** QCA outcomes by disclosure threshold using racial identity configurations (PLHIV only, n=17). Percentages shown for each burden indicator under Told3Plus/Told4Plus.

Told3+/4+	Informed Partners	Black	White	Family Rejects	Called Names	Unfair Work	Moved (Stigma)
0	0	1	0	50%/50%	50%/50%	17%/50%	50%/0%
1	1	1	0	67%/75%	83%/100%	67%/75%	33%/50%
1	1	0	1	50%/50%	100%/100%	100%/100%	100%/100%

Overall, the QCA results remain qualitatively stable under both thresholds, but the small subgroup sizes mean that the percentages should be interpreted cautiously. These findings suggest that racial identity interacts with disclosure behavior to shape reported social burden, and they highlight the need for larger studies to confirm these configurational patterns.

### Appendix A.4. Summary

Together, these robustness checks show that the overall interpretive structure of the typology and QCA findings is qualitatively stable, but sensitive to threshold selection in small samples. Under stricter thresholds for disclosure (Told4Plus) and social isolation, participants are redistributed into fewer typology categories, with most classified as either *Adaptive Disclosure* or *Concealed/Resilient*. However, the relative positioning of participants remains consistent: higher burden tends to cluster among participants with higher disclosure, while nondisclosure is not uniformly protective.

For the QCA analyses, using alternative disclosure thresholds produces modest shifts in subgroup percentages, but does not change the broader configurational patterns. Among sexual identity groups, straight participants with high disclosure consistently reported the highest burden, while LGB participants exhibited more variability depending on disclosure patterns. Among racial identity groups, White participants who disclosed widely reported the highest overall burden, but Black participants reported substantial exclusion even when disclosure was limited.

These findings reinforce two themes from the main analysis: (1) disclosure patterns interact with social identities to shape reported social burden in complex, context-dependent ways, and (2) subgroup sizes are small, so these results should be interpreted as exploratory and hypothesis-generating. Future studies with larger, more diverse samples will be needed to confirm these configurational patterns and assess how disclosure, social identity, and burden interact under different structural conditions.
